# Isolation of phages infecting the abundant freshwater *Actinobacteriota* order ‘*Ca*. Nanopelagicales’

**DOI:** 10.1038/s41396-023-01400-5

**Published:** 2023-03-24

**Authors:** Vinicius S. Kavagutti, Maria-Cecilia Chiriac, Rohit Ghai, Michaela M. Salcher, Markus Haber

**Affiliations:** 1 Department of Aquatic Microbial Ecology, Institute of Hydrobiology, Biology Centre CAS, Na Sádkách 702/7, 370 05 České Budějovice, Czech Republic; 2 Faculty of Science, University of South Bohemia, Branišovská 31, 370 05 České Budějovice, Czech Republic

**Keywords:** Bacteriophages, Virus-host interactions, Bacterial genomics, Water microbiology

## Abstract

Low-GC *Actinobacteriota* of the order ‘*Ca*. Nanopelagicales’ (also known as acI or hgcI clade) are abundant in freshwaters around the globe. Extensive predation pressure by phages has been assumed to be the reason for their high levels of microdiversity. So far, however, only a few metagenome-assembled phages have been proposed to infect them and no phages have been isolated. Taking advantage of recent advances in the cultivation of ‘*Ca*. Nanopelagicales’ we isolated a novel species of its genus ‘*Ca*. Planktophila’. Using this isolate as bait, we cultivated the first two phages infecting this abundant bacterial order. Both genomes contained a *whiB*-like transcription factor and a RNA polymerase sigma-70 factor, which might aid in manipulating their host’s metabolism. Both phages encoded a glycosyltransferase and one an anti-restriction protein, potential means to evade degradation of their DNA by nucleases present in the host genome. The two phage genomes shared only 6% of their genome with their closest relatives, with whom they form a previously uncultured family of actinophages within the *Caudoviricetes*. Read recruitment analyses against globally distributed metagenomes revealed the endemic distribution of this group of phages infecting ‘*Ca*. Nanopelagicales’. The recruitment pattern against metagenomes from the isolation site and the modular distribution of shared genes between the two phages indicate high levels of horizontal gene transfer, likely mirroring the microdiversity of their host in the evolutionary arms race between host and phage.

## Background

The numerical dominance of the *Actinobacteriota* order ‘*Ca*. Nanopelagicales’ in freshwaters worldwide has been attributed to their occurrence in very diversified habitat-specific consortia [1, 2]. Their microdiversity, together with potential high recombination rates, are thought to be a response to extensive phage predation levels [1]. This enables large population sizes despite high infection rates akin to the ‘king-of-the-mountain hypothesis’ proposed for marine SAR11 [3]. However, only a few metagenome-assembled phages have been proposed to infect this order [4, 5], mainly because of challenging in silico host prediction as their streamlined genomes do not encode CRISPR-cas systems [1, 6], whose spacers could be exploited for host-phage relationship prediction. ‘*Ca*. Nanopelagicales’ were for a long time uncultured or only transiently culturable [1, 6], but the recent identification of their heme auxotrophy now enables stable cultivation [7], allowing for culture-dependent phage isolation. Here we report the first cultivated phages infecting this order using a new ‘*Ca*. Nanopelagicales’ isolate as host.

## Host cultivation

We isolated strain LanE-43 from the Landštejn reservoir (Czech Republic) by dilution-to-extinction cultivation (see Supplementary Information). Genome-based analyses suggest it represents a novel species of ‘*Ca*. Planktophila’, for which we propose the name ‘*Ca*. P. landstejnensis’ (see Supplementary Information for details). As other ‘*Ca*. Nanopelagicales’ strains [1, 6] LanE-43 lacked a CRISPR-cas system (Supplementary Table S1), hence spacers cannot be used to identify its phages.

Based on read mapping from metagenomes (Supplementary Table S2), very similar ‘*Ca*. Nanopelagicales’ strains are present in the Řimov reservoir (Czech Republic) throughout the year and especially abundant in spring (April) in the epilimnion (0.5 m) (maximum coverage of 11.7× per Gbp metagenome).

## Phage isolation and genome analyses

**Fig. 1:**
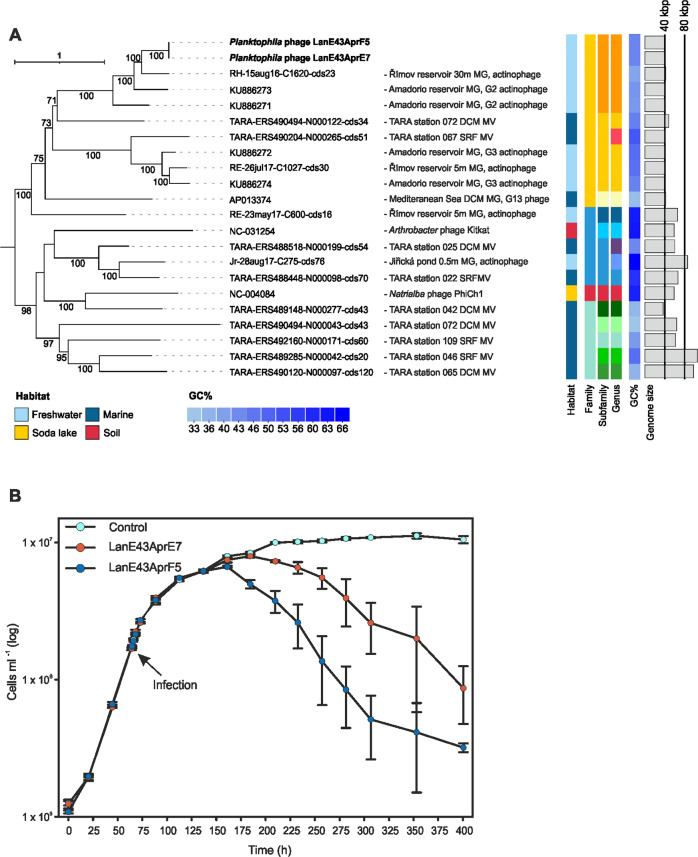
Phylogenetic analysis of the two isolated phages and their effect on the growth of their host LanE-43. **A** *terL* gene phylogenetic tree containing the isolated phages. For previously published genomes a description including the source is given (MG metagenome, MV metavirome, DCM deep chlorophyl maximum, SRF surface). Columns indicate habitat, whole genome taxonomic classification of clade members at the amino acid level using the Victor tool (identical colors indicate the same group at their respective level) [9], and GC% content. Bar plot: Genome size (scale bars are at 40 and 80 kbp). The entire tree is available as Supplementary Fig. S2. Bootstrap values >70% are indicated at the nodes. **B** Growth of the ‘*Ca*. Planktophila’ isolate LanE-43 and cultures infected with phages E7 and F5. An arrow indicates the time of infection. Dots represent the average LanE-43 abundance of three replicates ±standard error of the mean.

Given the distribution of LanE-43, we attempted phage isolation from the Řimov reservoir in April 2021 adapting protocols developed for the isolation of phages infecting marine fastidious heterotrophic bacteria [8]. Two phages, *Planktophila* phage LanE43AprE7 (from hereon E7) and *Planktophila* phage LanE43AprF5 (F5), able to infect and lyse LanE-43 (Fig. 1B) were successfully isolated and genome-sequenced.

Phylogenetic analyses of the large terminase subunit (*terL*; Fig. 1A) indicated that their closest relatives were two freshwater phages of the G2 clade from the Amadorio reservoir (Spain), proposed to infect a ‘*Ca*. Nanopelagicales’ host [4], and a proposed actinophage obtained from the Řimov reservoir [5]. These phage genomes had <70% ANI (average nucleotide identity) over alignment fractions of <6% to the two cultivated phages (Supplementary Table S3). Whole genome classification at the amino acid level [9] confirmed that the two cultured phages represent distinct species (Victor distance threshold >0.11898). According to our analysis, they represent a subfamily together with the three related phages [4, 5], which is part of a family of *Caudoviricetes* phages assembled from freshwater and marine metagenomes.

**Fig. 2:**
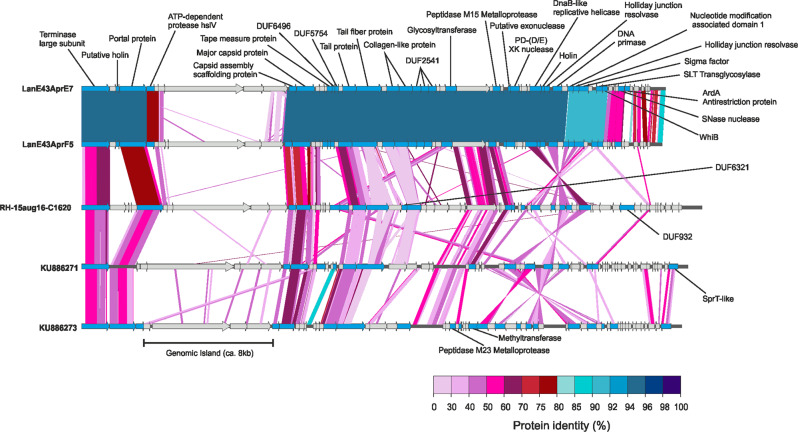
Genome comparison of the two isolated phages to their three closest relatives from the *terL* gene tree: RH-15aug16-C1620 from a metagenome of the Řimov reservoir from 30 m depth (ref. [5]), and two G2 actinophages (KU886271 and KU886273) from metagenomes of the Amadorio reservoir (ref. [4]). Annotated genes are highlighted in blue. Pairwise protein identity based on tBLASTx results is indicated by shaded areas. Phage genomes were aligned to start with the gene for the large terminase subunit.

Phages E7 and F5 had genome sizes of 39.1 kbp and 38.9 kbp, respectively, with a GC content (44.8% and 44.7%, respectively) slightly lower than their host (46.5%). E7 and F5 encoded 60 and 62 genes, respectively. Genome alignment showed synteny in most genes (Fig. 2). While the ANI between the two phages was 96.8%, the alignment fraction was only 63.6% of the shorter F5 genome, suggesting they are different viral species based on previously proposed thresholds (>95% ANI and >85% alignment fraction) [10]. The high sequence similarity in shared genes but low alignment fraction might indicate high gene flux [11]. The low alignment length was largely due to an apparent 8 kbp genomic island that encoded three hypothetical proteins in E7 and four in F5, which had no homologes in the other phage (Fig. 2).

Minor differences between E7 and F5 included a protein in E7 with a high probability (probability 99.51%, e-value 1.4E−13) HHpred hit to the ORF18 protein from *Enterococcus faecalis*. This protein is a homolog of the anti-restriction *ardA* gene and was shown to inhibit type I DNA restriction systems [12]. Hence, it might enable phage E7 to evade the restriction enzymes present in the host genome (Supplementary Table S1). All other genes, which we were able to annotate, were found in the genomes of both cultured phages (Fig. 2). Most were related to capsid biosynthesis, DNA packaging, and degradation of the host’s cell wall, proteins, and DNA (Supplementary Table S4). All members of the phage family containing E7 and F5 encode a *whiB*-like transcription factor, which is exclusively and universally found in *Actinobacteriota* [13] and suggested to be a key indicator for actinophages [4]. Phage-derived *whiB*-like proteins can prevent superinfection and inhibit cell septation and fragmentation [14].

Both cultivated phages also encoded an RNA polymerase sigma-70 factor (TIGR02943). These are frequently found in phage genomes and might be another way for the phage to control host gene transcription [4]. We also found glycosyltransferases in both phages (E7 and F5). These have been found previously in phages, including freshwater actinophages of the G2 and G3 clade [4], relatives of E7 and F5. One proposed role for phage-encoded glycosyltransferases is the protection of the phage genome against host nucleases by glycosylation [15]. This might be the case in E7 and F5 as their host, LanE-43, encodes several nucleases (Supplementary Table S1). Another potential role is the prevention of superinfection by modifying the cell wall glycosylation [15].

## Presence of phages E7 and F5 in metagenomes

Read mapping from metagenomes against the two phage genomes indicated that very similar phages (with reads covering >60% of the phage genome) are found only in the Řimov reservoir (Supplementary Table S2). Here they were present over multiple years, mainly in the epilimnion. This highly endemic distribution was also reported for the closest relatives, the G2 actinophages [4]. However, reads covering at least 30% of the phage genomes were found in lakes from the Czech Republic, Switzerland, Sweden, and the USA, suggesting that this phage family is widespread, like their host.

Recruitment plots can identify highly variable genome regions [16]. Two genomic islands were present in our phages (Supplementary Fig. S3): i) the 8 kbp island discussed above; ii) a region of 5 kbp that covered part of the tail fiber protein, two collagen-like proteins, and three proteins encoding DUF2541. As in marine phages [17], this second island might be related to host recognition.

Overall, the recruitment plots suggest that genomes of these actinophages are built up by three conserved genomic regions interspersed with two extremely variable regions. This pattern is suggestive of high horizontal gene transfer potentially due to extensive recombination in these variable regions as proposed for other aquatic bacteriophages [18]. The results described here are expected to boost further isolation efforts for such widely distributed host-phage systems in freshwaters and enable insights into the ecology of this widespread group of *Actinobacteriota*.

## Supplementary information

Supplemental Material

Supplementary Tables

## Data Availability

All genomes described here are deposited in ENA under the project accession number PRJEB57446. All sequence data used for alignments and phylogenetic trees is also available in FigShare (https://figshare.com/s/a223e0385e2832cee2a3).
