# Supplementary material for: Isolation of phages infecting the abundant freshwater Actinobacteriota order ‘Ca. Nanopelagicales’
Source: ISME J. 2023 Mar 24;17(6):943–6. doi: 10.1038/s41396-023-01400-5 (PMC10202952; doi:10.1038/s41396-023-01400-5)
Supplement: Supplementary file 1 — Supplemental Material [file 41396_2023_1400_MOESM1_ESM.pdf]

Supplementary Information for the article

Isolation of phages infecting the abundant freshwater *Actinobacteriota* order '*Ca.* Nanopelagicales'

By:

Vinicius S. Kavagutti, Maria-Cecilia Chiriac, Rohit Ghai, Michaela M. Salcher, Markus Haber

Content Page

Results

LanE-43 is a novel '*Ca*. Planktophila' species 2

Supplementary Figure S1: Phylogenomic tree of '*Ca*. Nanopelagicales' isolates 3

Supplementary Figure S2: Tree of the large subunit of phage terminase 4

Supplementary Figure S3: Recruitment plots 5

Methods

Host cultivation and genome sequencing 6

Phage isolation and genome sequencing 7

Genome assembly, annotation and comparisons 8

Reconstruction of phylogenetic trees 9

Recruitment analyses 9

References of Supplementary Material 10

Note: Supplementary Tables are presented in a separate file.

Results

*LanE-43 is a novel '*Ca*. Planktophila' species*

The taxonomic position of LanE-43 was inferred by a phylogenomic tree, average nucleotide identity (ANI), and GTDB classification. In the phylogenomic tree containing genomes of all previously available '*Ca*. Nanopelagicales' isolates, LanE-43 grouped within the well-supported clade of the genus '*Ca*. Planktophila' as a sister lineage to '*Ca*. Planktophila vernalis' (Neuenschwander et al. 2018) and unnamed species, the isolate IMCC19121 from Lake Soyang, South Korea (Kang et al. 2017) (Supplementary Fig. S1). ANI values to these two and all other genomes of '*Ca*. Nanopelagicales' isolates were <75% to all previously published genomes from '*Ca*. Nanopelagicales' isolates (Supplementary Table S5). Likewise, GTDB classification was only able to assign LanE-43 to the genus '*Ca*. Planktophila', with the closest species (GCA_001438925.1) failing to clear the predefine ANI radius inclusion as the same species (Supplementary Table S5). Given its taxonomic novelty and the availability of a complete genome, we propose to establish a novel species for isolate LanE-43 with the name '*Ca*. Planktophila landstejnensis' sp. nov. (land.stein.en'.sis N.L. fem. n. landstejnensis of or belonging to Landstein, named after the Landštejn reservoir (Czech Republic), from which LanE-43 was isolated).

As typical for members of the order '*Ca*. Nanopelagicales', LanE-43 had a small genome (1.43 Mbp) that was highly streamlined (coding density of 95.9%; median intergenic spacer of 10 bp) and had a low GC content (46.5%). The genome annotation to ascertain the absence of a CRISPR-cas system and identify nucleases is given in Supplementary Table S1.

While strains highly similar to LanE-43 were found in the Římov reservoir (Czech Republic), mapping of reads from additional metagenomes indicated that somewhat similar strains (reads covering 50-80% of the LanE-43 genome) can be found in lakes around the globe, usually at low abundance (<2x coverage per Gbp metagenome), but occasionally common (e.g., 9.7x coverage in Lake Loclat, Switzerland, in May 2009) (Supplementary Table S2).

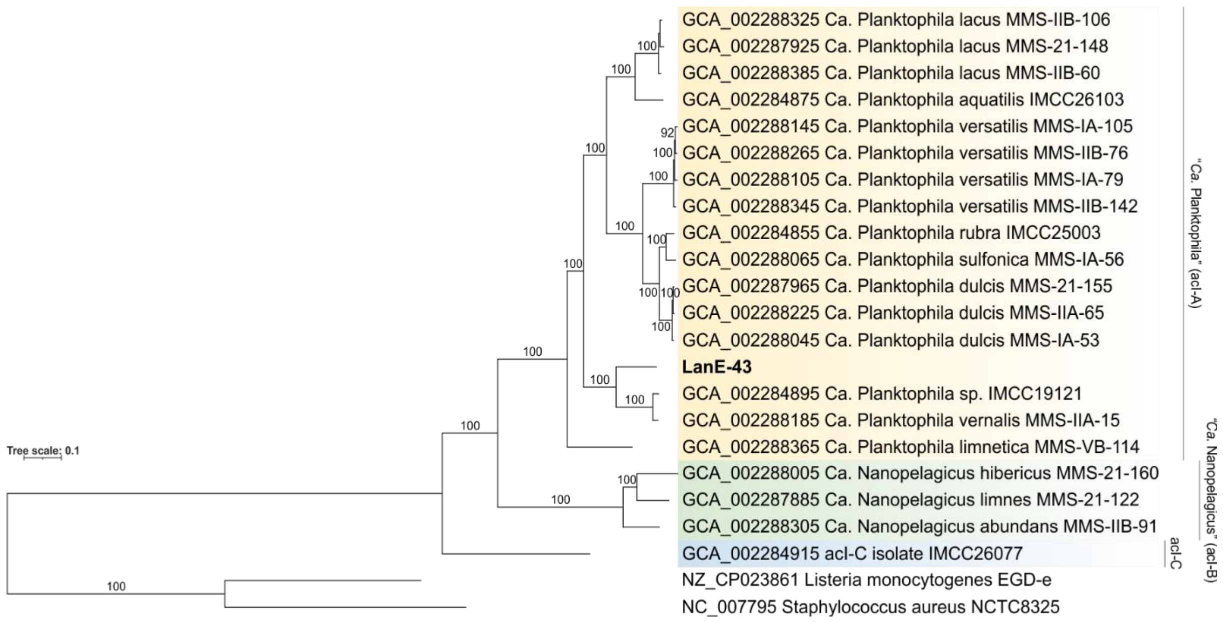

Supplementary Figure S1: Phylogenomic tree of strain LanE-43 and genomes of all previously available '*Ca*. Nanopelagicales'. The tree was rooted by the outgroup. Bootstrap values >70% are given at the nodes. For details, see methods below (page 9).

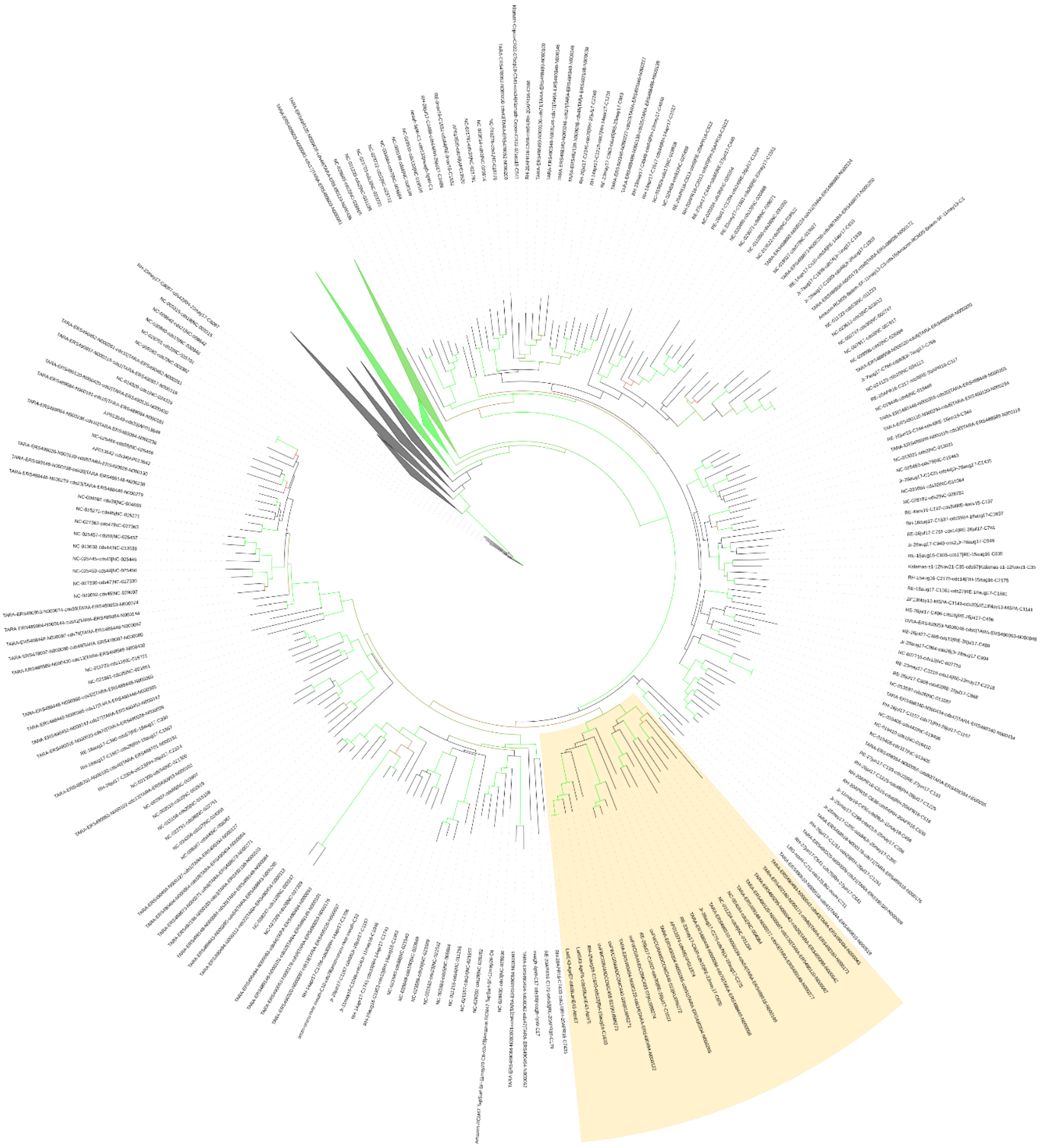

Supplementary Figure S2: Maximum likelihood tree of the *terL* gene (large subunit of the terminase) of a collection of 1050 marine, freshwater, and viral RefSeq phages. The clade visualized in Fig. 1 is highlighted (see below page 9 for details on the phage collection and methods). Branch colors indicate different levels of bootstrap support, from 90 (red) to 100% (green).

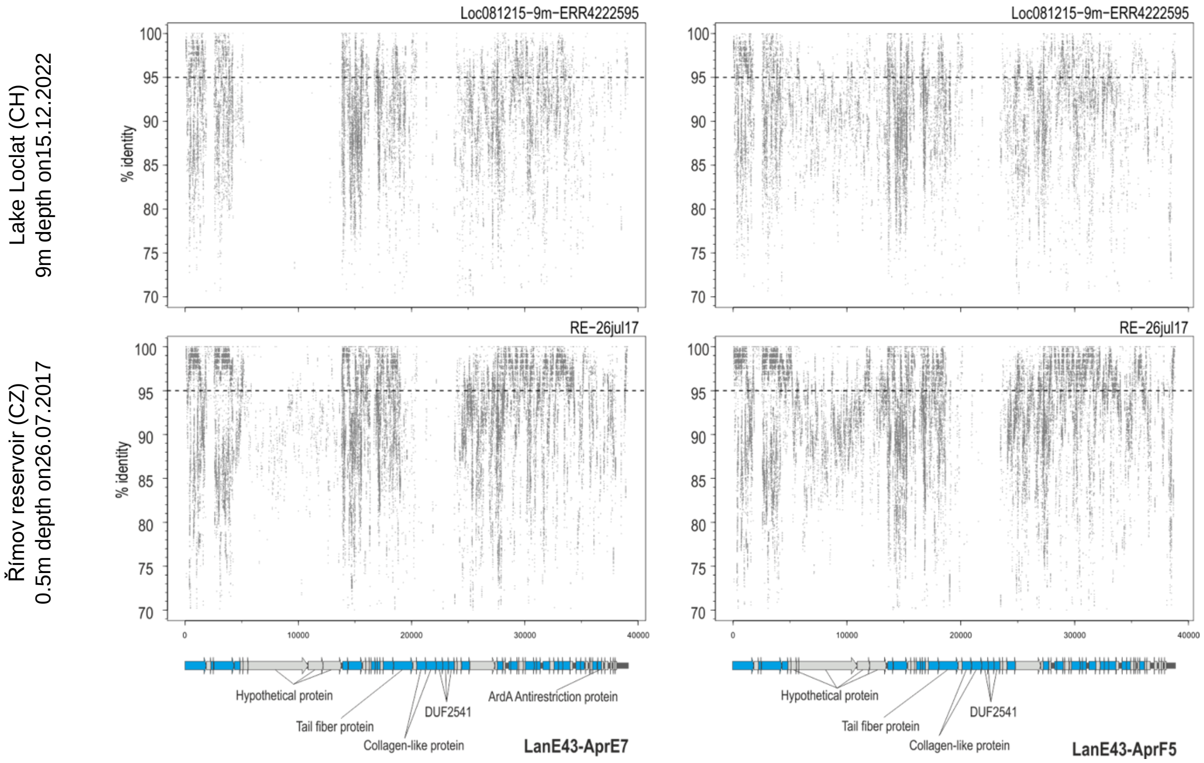

Supplementary Figure S3: Read recruitment of isolated phages LanE43AprE7 and LanE43AprF5 in the samples with the highest coverage per Gbp metagenome. Phage genomes are aligned below the graphs for orientation. Annotated genes are colored blue, and annotations of genes in genomic islands are given. Reads covered 49.0% and 57.9% of the genomes of E7 and F5, respectively, in Lake Loclat (CH) and 68.6% and 75.7%, respectively, in the Římov reservoir at 95% read identity (dashed lines). Note that these were not the highest percentages of the covered genome. The maximum was 89.5% and 91.8% in a sample from the Římov reservoir collected at 0.5m depth on May 23rd, 2017 (see Supplementary Table S3). Still, coverage per Gbp of metagenome was lower.

Methods

Host cultivation and genome sequencing

Isolation of planktonic *Actinobacteriota* followed previously established protocols (Neuenschwander *et al.* 2018) with slight modifications. All described culture incubations were performed at 16°C in a light:dark (16:8 h) cycle in medium 3 (see Supplementary Table S6 for medium composition), which is based on artificial lake water (Zotina, Köster and Jüttner 2003). 20 L water was collected from 5 m depth at the Landstejn reservoir (49°1'21'' N 15°14'30'' E, Czech Republic) on June 16th, 2020, and brought to the lab (ca. 1.5 hours after collection). A 15 ml water sample was 0.4 µm filtered, and cell abundance of the filtrate was determined by flow cytometry on a Beckman Coulter Cytoflex flow cytometer after staining cells with 1x SybrGreen. The filtrate was diluted in two steps and used to inoculate a 96 well plate filled with 1.5 ml media to give one row with 0.5 cells per well, 6 rows with 1 cell per well, and one row with 5 cells per well. After incubation for 6.5 weeks, the presence of growing bacteria was determined by flow cytometry. Strain LanE-43 originated from a well inoculated with 5 cells per well. 0.5 ml of the well content was transferred to a cryovial containing 250 µl 60% glycerol and frozen at -80°C for long-term stock preservation. The remaining well content was transferred to a sterile 14 ml cultivation tube filled with 4.5 ml medium for culture establishment. After 5 weeks, the tube was filled to 10 ml with fresh medium, and three weeks later, culture growth was determined by flow cytometry. 0.5 ml of the culture was transferred to 4.5 ml of fresh medium, and the culture was maintained following this 8-week cycle.

At the end of the first 8-week cultivation cycle, 2 ml of culture were pelleted by centrifugation (30 min at 14,000 rpm (20,817 x g^{-1}) in an Eppendorf 5430 centrifuge). 1.8 ml of the supernatant was discarded, and cells were lysed by two cycles of freezing at -80°C and thawing at 60°C. The lysate was used as a template to amplify the 16S rRNA gene (2 µl lysate, 7.5 µl ready mix, 5.5 µl molecular grade water, 1.5 µl of each 10 µM primer (27F and 1492r), PCR profile: 94 °C 3 min; 30 cycles of 94 °C 1 min, 52 °C 30 seconds, 72 °C 1 min 40 seconds; followed by 72 °C 10 min). The PCR product was purified with 0.15 µl of each FAST alkaline phosphatase and Exonuclease I (Thermo Scientific) at 37 °C for 45 min. The reaction was stopped by incubation at 85 °C for 30 min. 3 µl of the purified PCR product with 4.5 µl nuclease-free water and 2.5 µl of 10 µM primer 27 F were sent for Sanger sequencing to SEQme (Czech Republic). The sequence of strain LanE-43 indicated a pure culture and was identified as a member of the Actinobacteriota order '*Ca*. Nanopelagicales' (hgcI clade) by comparing the partial 16S rRNA gene sequence to the Silva database using the SILVA ACT service (www.arb-silva.de/aligner) and the SINA aligner v1.2.11 (Pruesse, Peplies and Glöckner 2012).

To obtain biomass for whole-genome sequencing, 400 ml medium was inoculated with strain LanE-43 and grown to stationary phase (ca. 1 x 10^7 cells/ml). The culture was filtered on a 0.22 µm filter using a peristaltic pump and the filter stored at -80°C until DNA extraction. The filter was cut into pieces and cells were lysed by bead beating with lysozyme using a 1:1 mix of sterile 1 and 0.7 mm diameter beads for 30 min at 1400 rpm followed by an additional 10 min after the addition of proteinase K. DNA was extracted with the Quick-DNA HMW MagBead kit (Zymo Research) following the manufacturer's instruction. The extracted DNA was sent to Novogene (Cambridge, UK) for library construction and sequencing using 2 x 150 bp pair-end reads on an Illumina NovaSeq 6000 instrument.

Phage isolation and genome sequencing

59 L water was collected from 0.5 m at the Římov reservoir (48°50'56" N 14°29'24" E, Czech Republic) on April 6th, 2021. Upon arrival at the lab (ca. 1 h after collection), the water was filtered sequentially through a 20 µm mesh, 5 µm, and 0.22 µm filters. The filtrate was stored overnight at 4°C before being concentrated by tangential flow filtration (TFF) (cutoff 100 kDA) to 1 L. The TFF concentrate was divided into two 500 ml aliquots and stored in sterile glass bottles in the dark at 4°C until further use.

One aliquot was used for phage isolation and purification following with slight modifications the protocol developed for fastidious heterotrophic bacteria (Buchholz *et al.* 2021), as LanE-43 like SAR11 and OM43 does not grow on agar plates. 12 wells of a 96 well plate were filled with 1 ml logarithmically growing LanE-43 culture (ca. 1 x 10^5 cells/ml), and 2 wells were filled with medium only. 100 µl of 0.1 µm filtered TFF concentrate was added to 8 wells with LanE-43 and one well with medium only. The two wells with LanE-43 only served as host growth controls, the medium-only wells as contamination controls of the medium, and the TFF concentrate. The inoculated plate was incubated for 3 weeks after which wells were screened by flow cytometry. Two of the 10 wells showing signs of infection (e.g., growth less than control wells, presence of cell debris) were selected for phage purification. The content of the wells was 0.1 µm filtered and kept at 4°C in sterile 2 ml Eppendorf tubes. For each phage, 11 wells of a 96 well plate were inoculated with 1 ml logarithmically growing LanE-43 and one well with medium only. 100 µl phage was added to the first well with LanE-43. The content was mixed and 100 µl transferred to the next well with LanE-43. This way, a serial dilution was performed for 10 wells with LanE-43 (10^{-1} to 10^{-10} dilutions). The last wells with LanE-43 only and the medium only served as controls. The plate was incubated for 3 weeks, growth was determined by flow cytometry, and the well with the highest dilution showing signs of infection was 0.1 um filtered and transferred to a sterile 2 ml Eppendorf and stored at 4°C. Two more purification rounds were performed in this way.

For phage genome sequencing, a 150 ml culture of mid-log-phase LanE-43 (ca. 1 x 10^6 cells ml^{-1}) was infected with 100 µl phage from the third purification. A control culture without phage was run in parallel. After confirming the completion of infection and lysis of the host via flow cytometry, a glycerol stock was prepared and kept at -80 °C. The phage-infected culture was 0.1 µm filtered, and a 10 ml aliquot was kept as stock at 4 °C. 100 ml lysate was used for phage DNA extraction using the PEG8000/NaCl protocol followed an established protocol (Buchholz *et al.* 2021). For each phage, two 50 ml Falcon tubes, each filled with 5 g polyethylenglycol (average molecular weight 8000) (Sigma Aldrich) and 3.3 g molecular grade NaCl (Sigma Aldrich), were filled to 50 ml with 0.1 µm filtered lysate. The salts were dissolved by inverting the tubes and the tubes were incubated overnight on ice. After centrifugation for 90 min at 4°C and 10000 x g (Beckman Coulter Avanti JXN-26 centrifuge with rotor JA-14.50), the supernatant was discarded. The viral pellets were resuspended in 1 ml SM buffer (100 mM NaCl, 8 mM $MgSO_4$ x 7 H_2O, 50 mM Tris-HCl) and the content of the two Falcon tubes were combined. DNA was extracted using the Wizard DNA Clean-Up System kit (Promega) following the manufacturer's instructions but using 60°C preheated nuclease-free water for DNA elution. The first elution of the two columns used per phage were pooled, and the extracted DNA was sent to Novogene (Cambridge, UK) for library construction, and genome sequencing using 2 x 150 bp pair-end reads on an Illumina NovaSeq 6000 instrument.

Genome assembly, annotation, and comparisons

Raw sequences were quality filtered, and the Illumina adaptors together with the phiX and p-Fosil2 control reads were removed using the BBMap project tools (https://github.com/BioInfoTools/BBMap/) with the bbduk.sh script run using the following parameters: qtrim = rl, trimq = 18, k = 21, ref = adapterfile ordered cardinality. The processed reads were assembled with SPAdes (Bankevich *et al.* 2012) with the following k-mer list: 29, 49, 59, 69, 79, 89, 99, 109, 119, 127. Quality filtered reads were mapped onto the assemblies (1 contig) in Geneious Prime 2022.1.1 (www.geneious.com), resulting in overlapping ends that allowed the circularization of genomes. The LanE-43 genome was set to start with the *dnaA* gene. The phage genomes were set to start with the *terL* gene to ease comparison between the phages.

Genes were predicted with Prodigal (Hyatt *et al.* 2010). For the LanE-43 genome, all the predicted protein domains coding sequences were scanned with Interproscan (Quevillon *et al.* 2005; Jones *et al.* 2014). The sequences were scanned locally using the HMMER3 package (Eddy 2011) against Clusters of Orthologous Group (COGs) (Tatusov *et al.* 2003), TIGR Families—TIGRfams (e-values = 1e-3) (Haft 2001) and KOALA algorithm against a non-redundant KEGG genes database (Kanehisa, Sato and Morishima 2016). The script pfam_scan.pl (ftp.sanger.ac.uk/pub/databases/Pfam/Tools/) was also used to identify Pfam domains with the PFAM database release 35 (Mistry *et al.* 2021). Predicted protein sequences of the phage genomes were manually annotated using hhpred (Zimmermann *et al.* 2018) and the following four databases: PDB_mmCIF70_12_Aug, UniProt-SwissProt-viral70_3_Nov_2021, NCBI_Conserved_Domains(CD)_v3.19, Pfam-A v35. Searches were run with the following parameters: MSA generation method – Hhblits=>UniRef30, MSA generation iterations – 3, E-value cutoff for MSA generation – 1e-3, Min seq identity of MSA hits with query (%) – 0, Min coverage of mSA hits (%) – 20, Secondary structure scoring – during alignment, Alignment Mode: Realign with MAC – local: norealign, MAC realignment threshold – 0.3, Max target hits 250, Min probability in hitlist (%) – 20. The top hits and the top hits with functional annotation, are reported in Supplementary Table S4. In general, only hits with >95% probability were considered for annotation. Annotations for hits with a probability between 90 and 95% were accepted on a case-by-case basis (e.g., known highly variable genes were considered). For this reason, an additional annotation was conducted using the script pfam_scan.pl (ftp://ftp.sanger.ac.uk/pub/databases/Pfam/Tools/) to identify Pfam domains with the PFAM database release 35 (Mistry *et al.* 2021) and functional domains were identified using the Conserved Domain Database (CDD) (Lu *et al.* 2020).

Average nucleotide identity (ANI) between the isolate LanE-43 and all previously isolated '*Ca*. Nanopelagicales' genomes was calculated using the method described by (Goris et al. 2007). GTDB classification was done usingGTDB-Tk (version 2.1) (Parks *et al.* 2018; Chaumeil *et al.* 2020). For the phages, ANI and alignment fraction were calculated between the two phages and the closest three phages of the *terL* gene tree in the same way. Whole genome classification of phages LanE43AprE7 and LanE43AprF5 and their closest relatives based on *terL* phylogeny was performed with the VICTOR tool (available at: https://victor.dsmz.de) at the amino acid level using the D6 formula (Meier-Kolthoff and Göker 2017). Similarity plots of phage genomes were created using the Flex2 program (available at: https://github.com/asierzaragoza/flex2) using TBLASTX, BLOSUM45 matrix, minimum identity 30%, and minimum 55 amino acid alignment.

Reconstruction of phylogenetic trees

The phylogenetic tree of the host LanE-43 was reconstructed using genomes of all publicly available '*Ca*. Nanopelagicales' isolates (16 '*Ca*. Planktophila' genomes, 3 '*Ca*. Nanopelagicus' genomes and the genome of the acI-C isolate IMCC26077). The genomes of *Staphylococcus aureus* NCTC8325 and Listeria monocytogenes EGDe were included as outgroup. The tree was based on 120 bacterial single-copy marker genes (Parks *et al.* 2018), individually aligned using PRANK (Löytynoja 2014). The alignments were trimmed using BMGE (-m BLOSUM62 -t AA -g 0.5 -b 5) (Criscuolo and Gribaldo 2010) and concatenated. A Maximum-likelihood phylogeny was generated with IQ-TREE2 (-bb 1000, -alrt 1000) (Minh *et al.* 2020) and the LG + F + G4 evolutionary model, which was determined by ModelFinder as the best model for the data (Kalyaanamoorthy *et al.* 2017).

For the phage *terL* gene tree, a collection of 5480 genomes containing marine (Mizuno *et al.* 2013; Brum *et al.* 2015; Nishimura *et al.* 2017) and freshwater (Ghai *et al.* 2017; Kavagutti *et al.* 2019) as well as NCBI Viral RefSeq phages, was scanned locally using HMMER3 package against the Evolutionary Classification of Protein Domains (ECOD209) (Cheng *et al.* 2014) for the large subunit of the terminase gene (ID domains: E1W9HA2, E4DKWA1, E4DKWD1, E4DKWC1) using a minimum probability of 70% and a minimum hit coverage of 70 amino acids. Potential hits were annotated as described above for the LanE-43 genome to confirm the hit as terminase. Sequences smaller than 150 amino acids were removed. The resulting 4943 *bona fide* terminase sequences were dereplicated using MMseqs2 with the easy-cluster option (Steinegger and Söding 2017), resulting in 1050 dereplicated terminase amino acid sequences. These were aligned using muscle v5 (-super5 option) (Edgar 2022). The alignment was used to construct a maximum-likelihood tree using IQ-TREE2 with 1,000 iterations of ultrafast bootstrapping (Hoang *et al.* 2018), SH testing (Minh *et al.* 2020), and a restricted model selection of sequence evolution (-mset "WAG,LG,JTT,JTTDCMut,Dayhoff,DCMut,VT,PMB").

The final trees were visualized with iTOL (http://itol.embl.de) (Letunic and Bork 2016).

Recruitment analyses and plots

Before the recruitment analysis for the LanE-43 genome, we identified and masked the ribosomal RNA genes (5S,16S, and 23S rRNA) using Prokka (Seemann 2014) to avoid biases when computing the abundances. Reads from 499 freshwater metagenomic datasets (Supplementary Table S3) were then mapped against the LanE-43 genome and the two phage genomes using RazerS 3 (Weese, Holtgrewe and Reinert 2012) using a minimum identity of 95% and a minimum alignment length of 50 bp as thresholds. Coverage per Gbp of metagenome and percentage of genome covered were recorded (Supplementary Table S2). For selected metagenomes, recruitment plots (Supplementary Fig. S3) were made based on read mapping with a minimum identity 70% and minimum alignment length 50 bp.

References

Bankevich A, Nurk S, Antipov D *et al.* SPAdes: a new genome assembly algorithm and its applications to single-cell sequencing. *J Comput Biol* 2012;**19**:455–77.

Brum JR, Ignacio-Espinoza JC, Roux S *et al.* Patterns and ecological drivers of ocean viral communities. *Science* 2015;**348**:1261498.

Buchholz HH, Michelsen ML, Bolaños LM *et al.* Efficient dilution-to-extinction isolation of novel virus–host model systems for fastidious heterotrophic bacteria. *ISME J* 2021;**15**:1585–98.

Chaumeil P-A, Mussig AJ, Hugenholtz P *et al.* GTDB-Tk: a toolkit to classify genomes with the Genome Taxonomy Database. *Bioinformatics* 2020;**36**:1925–7.

Cheng H, Schaeffer RD, Liao Y *et al.* ECOD: An evolutionary classification of protein domains. *PLoS Comput Biol* 2014;**10**.

Criscuolo A, Gribaldo S. BMGE (block mapping and gathering with entropy): a new software for selection of phylogenetic informative regions from multiple sequence alignments. *BMC Evol Biol* 2010;**10**:210.

Eddy SR. Accelerated profile HMM searches. *PLoS Comput Biol* 2011;**7**:e1002195.

Edgar RC. High-accuracy alignment ensembles enable unbiased assessments of sequence homology and phylogeny. *bioRxiv* 2022.

Ghai R, Mehrshad M, Mizuno CM *et al.* Metagenomic recovery of phage genomes of uncultured freshwater actinobacteria. *ISME J* 2017;**11**:304–8.

Goris J, Konstantinidis KT, Klappenbach JA *et al.* DNA-DNA hybridization values and their relationship to whole-genome sequence similarities. *Int J Syst Evol Microbiol* 2007;**57**:81–91.

Haft DH. TIGRFAMs: a protein family resource for the functional identification of proteins. *Nucleic Acids Res* 2001;**29**:41–3.

Hoang DT, Chernomor O, von Haeseler A *et al.* UFBoot2: improving the ultrafast bootstrap approximation. *Mol Biol Evol* 2018;**35**:518–22.

Hyatt D, Chen G-L, LoCascio PF *et al.* Prodigal: prokaryotic gene recognition and translation initiation site identification. *BMC Bioinformatics* 2010;**11**:119.

Jones P, Binns D, Chang H-Y *et al.* InterProScan 5: genome-scale protein function classification. *Bioinformatics* 2014;**30**:1236–40.

Kalyaanamoorthy S, Minh BQ, Wong TKF *et al.* ModelFinder: fast model selection for accurate phylogenetic estimates. *Nat Methods* 2017;**14**:587–9.

Kanehisa M, Sato Y, Morishima K. BlastKOALA and GhostKOALA: KEGG Tools for functional characterization of genome and metagenome sequences. *J Mol Biol* 2016;**428**:726–31.

Kang I, Kim S, Islam MdR *et al.* The first complete genome sequences of the acI lineage, the most abundant freshwater Actinobacteria, obtained by whole-genome-amplification of dilution-to-extinction cultures. *Sci Rep* 2017;**7**:42252.

Kavagutti VS, Andrei A-Ş, Mehrshad M *et al.* Phage-centric ecological interactions in aquatic ecosystems revealed through ultra-deep metagenomics. *Microbiome* 2019;**7**:135.

Letunic I, Bork P. Interactive tree of life (iTOL) v3: an online tool for the display and annotation of phylogenetic and other trees. *Nucleic Acids Res* 2016.

Löytynoja A. Phylogeny-aware alignment with PRANK. In: Russell DJ (ed.). *Multiple Sequence Alignment Methods*. Totowa, NJ: Humana Press, 2014, 155–70.

Lu S, Wang J, Chitsaz F *et al.* CDD/SPARCLE: the conserved domain database in 2020. *Nucleic Acids Res* 2020;**48**:D265–8.

Meier-Kolthoff JP, Göker M. VICTOR: genome-based phylogeny and classification of prokaryotic viruses. *Bioinformatics* 2017;**33**:3396–404.

Minh BQ, Schmidt HA, Chernomor O *et al.* IQ-TREE 2: New models and efficient methods for phylogenetic inference in the genomic era. *Mol Biol Evol* 2020;**37**:1530–4.

Mistry J, Chuguransky S, Williams L *et al.* Pfam: The protein families database in 2021. *Nucleic Acids Res* 2021;**49**:D412–9.

Mizuno CM, Rodriguez-Valera F, Kimes NE *et al.* Expanding the marine virosphere using metagenomics. *PLOS Genet* 2013;**9**:e1003987.

Neuenschwander SM, Ghai R, Pernthaler J *et al.* Microdiversification in genome-streamlined ubiquitous freshwater Actinobacteria. *ISME J* 2018;**12**:185–98.

Nishimura Y, Watai H, Honda T *et al.* Environmental viral genomes shed new light on virus-host interactions in the ocean. *mSphere* 2017;**2**:e00359-16.

Parks DH, Chuvochina M, Waite DW *et al.* A standardized bacterial taxonomy based on genome phylogeny substantially revises the tree of life. *Nat Biotechnol* 2018;**36**:996–1004.

Pruesse E, Peplies J, Glöckner FO. SINA: Accurate high-throughput multiple sequence alignment of ribosomal RNA genes. *Bioinformatics* 2012;**28**:1823–9.

Quevillon E, Silventoinen V, Pillai S *et al.* InterProScan: protein domains identifier. *Nucleic Acids Res* 2005;**33**:W116–20.

Seemann T. Prokka: Rapid prokaryotic genome annotation. *Bioinformatics* 2014;**30**:2068–9.

Steinegger M, Söding J. MMseqs2 enables sensitive protein sequence searching for the analysis of massive data sets. *Nat Biotechnol* 2017.

Tatusov RL, Fedorova ND, Jackson JD *et al.* The COG database: an updated version includes eukaryotes. *BMC Bioinformatics* 2003;**4**:41.

Weese D, Holtgrewe M, Reinert K. RazerS 3: faster, fully sensitive read mapping. *Bioinformatics* 2012;**28**:2592–9.

Zimmermann L, Stephens A, Nam S-Z *et al.* A completely reimplemented MPI bioinformatics toolkit with a new HHpred server at its core. *J Mol Biol* 2018;**430**:2237–43.

Zotina T, Köster O, Jüttner F. Photoheterotrophy and light-dependent uptake of organic and organic nitrogenous compounds by *Planktothrix rubescens* under low irradiance. *Freshw Biol* 2003;**48**:1859–72.
